# Supplementary material for: HIVprotI: an integrated web based platform for prediction and design of HIV proteins inhibitors
Source: J Cheminform. 2018 Mar 9;10:12. doi: 10.1186/s13321-018-0266-y (PMC5845081; doi:10.1186/s13321-018-0266-y)
Supplement: Supplementary file 2 — Additional file 2. Source code of HIVProtI web server. [file 13321_2018_266_MOESM2_ESM.zip › HIVprotI_Source-code/hivprotI/weka-3-6-6/doc/weka/gui/beans/BeanInstance.html]

BeanInstance


---


|  |  |  |
| --- | --- | --- |
| **Overview** \| **Package** \| **Class** \| **Tree** \| **Deprecated** \| **Index** \| **Help** \| **Weka's home** | |  |
| **PREV CLASS**   **NEXT CLASS** | **FRAMES**    **NO FRAMES**     **All Classes** |  |
| SUMMARY: NESTED \| FIELD \| CONSTR \| METHOD | DETAIL: FIELD \| CONSTR \| METHOD |  |


---


## weka.gui.beans Class BeanInstance

```
java.lang.Object
  weka.gui.beans.BeanInstance
```

**All Implemented Interfaces:**: java.io.Serializable

---

``` public class BeanInstance extends java.lang.Object implements java.io.Serializable ```

Class that manages a set of beans.

**Since:**
:   1.0

**Version:**
:   $Revision: 7059 $

**Author:**
:   Mark Hall

**See Also:**: Serialized Form

---

| **Field Summary** | |
| --- | --- |
| `static int` | `BEAN_EXECUTING` |
| `static int` | `IDLE` |


**Constructor Summary**

`BeanInstance(javax.swing.JComponent container,
java.lang.Object bean,
int x,
int y)`
  
          Creates a new `BeanInstance` instance.

`BeanInstance(javax.swing.JComponent container,
java.lang.String beanName,
int x,
int y)`
  
          Creates a new `BeanInstance` instance given the fully
qualified name of the bean


| **Method Summary** | |
| --- | --- |
| `static void` | `addAllBeansToContainer(javax.swing.JComponent container)`             Adds all beans to the supplied component |
| `void` | `addBean(javax.swing.JComponent container)`             Adds this bean to the global list of beans and to the supplied container. |
| `static BeanInstance` | `findInstance(java.awt.Point p)`             Looks for a bean (if any) whose bounds contain the supplied point |
| `static java.util.Vector` | `findInstances(java.awt.Rectangle boundingBox)`             Looks for all beans (if any) located within the supplied bounding box. |
| `java.lang.Object` | `getBean()`             Gets the bean encapsulated in this instance |
| `static java.util.Vector` | `getBeanInstances()`             Return the list of displayed beans |
| `int` | `getHeight()`             Gets the height of this bean |
| `int` | `getWidth()`             Gets the width of this bean |
| `int` | `getX()`             Gets the x coordinate of this bean |
| `int` | `getY()`             Gets the y coordinate of this bean |
| `static void` | `paintLabels(java.awt.Graphics gx)`             Renders the textual labels for the beans. |
| `static void` | `removeAllBeansFromContainer(javax.swing.JComponent container)`             Removes all beans from containing component |
| `void` | `removeBean(javax.swing.JComponent container)`             Remove this bean from the list of beans and from the containing component |
| `static void` | `reset(javax.swing.JComponent container)`             Reset the list of beans |
| `static void` | `setBeanInstances(java.util.Vector beanInstances, javax.swing.JComponent container)`             Describe `setBeanInstances` method here. |
| `void` | `setX(int newX)`             Sets the x coordinate of this bean |
| `void` | `setXY(int newX, int newY)`             Set the x and y coordinates of this bean |
| `void` | `setY(int newY)`             Sets the y coordinate of this bean |

**Methods inherited from class java.lang.Object**

`equals, getClass, hashCode, notify, notifyAll, toString, wait, wait, wait`

**Field Detail**

### IDLE

```
public static final int IDLE
```

**See Also:**: Constant Field Values

---


### BEAN\_EXECUTING

```
public static final int BEAN_EXECUTING
```

**See Also:**: Constant Field Values


**Constructor Detail**

### BeanInstance

```
public BeanInstance(javax.swing.JComponent container,
                    java.lang.Object bean,
                    int x,
                    int y)
```

:   Creates a new `BeanInstance` instance.

    **Parameters:**: `container` - a `JComponent` to add the bean to: `bean` - the bean to add: `x` - the x coordinate of the bean: `y` - the y coordinate of the bean

---


### BeanInstance

```
public BeanInstance(javax.swing.JComponent container,
                    java.lang.String beanName,
                    int x,
                    int y)
```

:   Creates a new `BeanInstance` instance given the fully
    qualified name of the bean

    **Parameters:**: `container` - a `JComponent` to add the bean to: `beanName` - the fully qualified name of the bean: `x` - the x coordinate of the bean: `y` - th y coordinate of the bean


**Method Detail**

### reset

```
public static void reset(javax.swing.JComponent container)
```

:   Reset the list of beans

---


### removeAllBeansFromContainer

```
public static void removeAllBeansFromContainer(javax.swing.JComponent container)
```

:   Removes all beans from containing component

    :   **Parameters:**: `container` - a `JComponent` value

---


### addAllBeansToContainer

```
public static void addAllBeansToContainer(javax.swing.JComponent container)
```

:   Adds all beans to the supplied component

    :   **Parameters:**: `container` - a `JComponent` value

---


### getBeanInstances

```
public static java.util.Vector getBeanInstances()
```

:   Return the list of displayed beans

    :   **Returns:**: a vector of beans

---


### setBeanInstances

```
public static void setBeanInstances(java.util.Vector beanInstances,
                                    javax.swing.JComponent container)
```

:   Describe `setBeanInstances` method here.

    :   **Parameters:**: `beanInstances` - a `Vector` value: `container` - a `JComponent` value

---


### paintLabels

```
public static void paintLabels(java.awt.Graphics gx)
```

:   Renders the textual labels for the beans.

    :   **Parameters:**: `gx` - a `Graphics` object on which to render the labels

---


### findInstance

```
public static BeanInstance findInstance(java.awt.Point p)
```

:   Looks for a bean (if any) whose bounds contain the supplied point

    :   **Parameters:**: `p` - a point **Returns:**: a bean that contains the supplied point or null if no bean contains the point

---


### findInstances

```
public static java.util.Vector findInstances(java.awt.Rectangle boundingBox)
```

:   Looks for all beans (if any) located within
    the supplied bounding box. Also adjusts the
    bounding box to be a tight fit around all
    contained beans

    :   **Parameters:**: `boundingBox` - the bounding rectangle **Returns:**: a Vector of BeanInstances

---


### removeBean

```
public void removeBean(javax.swing.JComponent container)
```

:   Remove this bean from the list of beans and from the containing component

    :   **Parameters:**: `container` - the `JComponent` that holds the bean

---


### addBean

```
public void addBean(javax.swing.JComponent container)
```

:   Adds this bean to the global list of beans and
    to the supplied container. The constructor
    calls this method, so a client should not need
    to unless they have called removeBean and then
    wish to have it added again.

    :   **Parameters:**: `container` - the Component on which this BeanInstance will be displayed

---


### getBean

```
public java.lang.Object getBean()
```

:   Gets the bean encapsulated in this instance

    :   **Returns:**: an `Object` value

---


### getX

```
public int getX()
```

:   Gets the x coordinate of this bean

    :   **Returns:**: an `int` value

---


### getY

```
public int getY()
```

:   Gets the y coordinate of this bean

    :   **Returns:**: an `int` value

---


### getWidth

```
public int getWidth()
```

:   Gets the width of this bean

    :   **Returns:**: an `int` value

---


### getHeight

```
public int getHeight()
```

:   Gets the height of this bean

    :   **Returns:**: an `int` value

---


### setXY

```
public void setXY(int newX,
                  int newY)
```

:   Set the x and y coordinates of this bean

    :   **Parameters:**: `newX` - the x coordinate: `newY` - the y coordinate

---


### setX

```
public void setX(int newX)
```

:   Sets the x coordinate of this bean

    :   **Parameters:**: `newX` - an `int` value

---


### setY

```
public void setY(int newY)
```

:   Sets the y coordinate of this bean

    :   **Parameters:**: `newY` - an `int` value


---


|  |  |  |
| --- | --- | --- |
| **Overview** \| **Package** \| **Class** \| **Tree** \| **Deprecated** \| **Index** \| **Help** \| **Weka's home** | |  |
| **PREV CLASS**   **NEXT CLASS** | **FRAMES**    **NO FRAMES**     **All Classes** |  |
| SUMMARY: NESTED \| FIELD \| CONSTR \| METHOD | DETAIL: FIELD \| CONSTR \| METHOD |  |


---
